# Supplementary material for: Relationship between Treatment Plan Dosimetry, Toxicity, and Survival following Intensity-Modulated Radiotherapy, with or without Chemotherapy, for Stage III Inoperable Non-Small Cell Lung Cancer
Source: Cancers (Basel). 2021 Nov 25;13(23):5923. doi: 10.3390/cancers13235923 (PMC8657053; doi:10.3390/cancers13235923)
Supplement: Supplementary file 1 [file cancers-13-05923-s001.zip › cancers-1414492-supplementary.pdf]

cancers MDPI

Table S1. Overview of toxicities reported in other studies.

REF	CTCAE version	CCRT	CCRT + IMT	SCRT	Radical RT
**Pneumonitis ≥ 2**					
Our analysis	5.0	25.0%		17% (scrt/rt)	
Palma, IJROBP, 2013- Meta-analysis	-	29.8%			
Liang, Ann Oncol, 2017	-	18.9–33.3%			
Wang, Lung Cancer, 2012	Nci ctc 2.0	25%			
**Pneumonitis grade 2**					
Our analysis	5.0	23%		14% (scrt/rt)	
**Phase II trials**					
Wang, Lung Cancer, 2012	Nci ctc 2.0	18.1%			
**Phase III trials**					
RTOG 0617, Lancet Oncol, 2015	3.0	4%			
**"Real world"**					
Naidoo, JCO, 2017. Different types of cancers	4.0				
**Pneumonitis grade ≥3**					
Our analysis	5.0	2%		3% (scrt/rt)	
**Phase II trials**					
ETOP NICOLAS trial, Lung Cancer, 2019			0%		
PET boost trial, Radiother Oncol, 2019	3.0	0%		6.7%	
Wang, Lung Cancer, 2012	Nci ctc 2.0	6.1%			
**Phase III Trials**					
Antonia, NEJM, 2017	?	3.8% (1.3% Gr5)	4.2% (0.8% Gr5)		

RTOG-9410, J Natl Cancer Inst, 2011<br>Acute<br>Late		 2.1–3.6%<br>12.5–14.0%		 8.7%<br>14.8%	
EORTC 08972-22973, Eur J Cancer, 2007		18%	14%		
NPC 95-01, J Clin Oncol, 2005		5%	11%		
RTOG 0617, Lancet Oncol, 2015	3.0	4.7%			
JCOG0301, Lancet Oncol, 2012		1.0%			3.1%
**"Real world"**					
Wiersma, Lung Cancer, 2013	4.03	4.1%			
Phernambucq, Ann Oncol, 2010		7.9%			
Naidoo, JCO, 2017. Different types of cancers	4.0				
Dieleman, IJROBP, 2018	4.0	1.3%			
Kim, Radiat Oncol J, 2019	4.0	18.5%			14.3%
Jeremic, IJROBP, 2012		12.1%			5.5%
**Esophagitis grade ≥2**					
Our analysis	5.0	40.0%		48% (scrt/rt)	
Kim, Radiat Oncol J, 2019	4.0	44.4%			17.9%
Wang, Lung Cancer, 2012 (Ph II)	Nci ctc 2.0	37.5%			
**Esophagitis grade 2**					
Our analysis	5.0	23%		37% (scrt/rt)	
Palma, IJROBP, 2013- Meta-analysis		32.2%			
**Phase III trials**					
RTOG 0617, Lancet Oncol, 2015	3.0	23–24%			
**"Real world"**					
**Dysphagia/Esophagitis grade ≥3**					
Our analysis	5.0	17%		11% (scrt/rt)	

Palma, IJROBP, 2013- Meta-analysis		18.0%			
**Phase II Trials**					
PET boost trial, Radiother Oncol, 2019	3.0	14.3%		3.3%	
**Phase III trials**					
EORTC 08972-22973, Eur J Cancer, 2007		14%	5%		
RTOG-9410, J Natl Cancer Inst, 2011		22–45%		4%	
NPC 95-01, J Clin Oncol, 2005		32%	3%		
Blanke, J Clin Oncol, 1995		3%			
RTOG 0617, Lancet Oncol, 2015	3.0	3–7%			
Liang, Ann Oncol, 2017		6.3–20.0%			
**"Real world"**					
Wiersma, Lung Cancer, 2013	4.03	33.9%			
Phernambucq, Ann Oncol, 2010		28.1%			
Dieleman, IJROBP, 2018	4.0	8.4%			
Jeremic, IJROBP, 2012		10.6%			4.7%
**Cardiac events**					
Our analysis	5.0	8%		17% (scrt/rt)	
**Phase II trials**					
PET boost trial, Radiother Oncol, 2019	3.0	6.5% (≥3)		3.3% (≥3)	
**Phase III trials**					
RTOG 0617, Lancet Oncol, 2015	3.0	11%			
**"Real world"**					
Dess, JCO, 2017. St II-III					
Dieleman, IJROBP, 2018	4.0	2.6% (≥3)			
Jeremic, IJROBP, 2012	-	0.2% (≥3)			0%

Table S2. Univariate analysis for progression free survival.

PTV per 100cc increase; Mean lung dose per 100 cGy increase; V25Gy/V40Gy/V60Gy heart per 10% increase; V5Gy contralateral lung per 10% increase;

	cCRT			sCRT/RT		
	**HR**	**95% CI**	**p-value**	**HR**	**95% CI**	**P-value**
V25Gy heart (%)	1.14	0.94–1.38	0.19	0.91	0.76–1.08	0.27
V40Gy heart (%)	1.20	0.97–1.49	0.10	0.87	0.70–1.08	0.20
V60Gy heart (%)	1.35	0.96–1.88	0.08	0.89	0.63–1.17	0.34
Mean heart dose (cGy)	1.02	0.99–1.11	0.12	0.98	0.95–1.00	0.27
V20Gy lungs (%)	1.19	0.70–2.04	0.52	1.20	0.87–1.64	0.27
V25Gy lungs (%)	1.17	0.67–2.06	0.59	1.20	0.85–1.69	0.30
V5Gy contralateral lung (%)	1.20	1.00–1.44	0.06	1.08	0.89–1.31	0.43
Mean lung dose (cGy)	1.03	0.94–111	0.49	1.02	0.97–1.11	0.40
V20Gy esopahgus (cm^3)	1.01	0.99–1.04	0.26	1.00	0.97–1.02	0.72
V40Gy esopahgus (cm^3)	1.02	0.99–1.05	0.14	1.00	0.97–1.02	0.77
V50Gy esopahgus (cm^3)	1.03	1.00–1.06	0.06	0.99	0.97–1.02	0.68
V60Gy esopahgus (cm^3)	1.03	0.99–1.07	0.21	0.99	0.96–1.03	0.71
N-stage 2	0.65	0.22–1.92	0.44	0.88	0.26–30.4	0.84
N-stage 3	0.86	0.27–2.72	0.80	1.01	0.28–3.59	0.99
Technique full VMAT	0.95	0.46–1.95	0.87	0.97	0.52–1.82	0.93
Age	1.02	0.98–1.06	0.25	1.02	0.99–1.04	0.23
Smoking history	1.01	1.00–1.02	0.15	1.01	1.00–1.02	0.16
PTV volume	1.18	1.11–1.35	0.01	0.98	0.91–1.11	0.57

Abbreviations: V25Gy/V40Gy/V60Gy heart: percentage of the heart that received at least 25/40/60Gy; V20Gy/V20Gy lungs: percentage of the total lung minus PTV that received at least 20/40/50Gy; V5Gy contralateral lung: percentage of the contralateral lung that received at least 5 Gy; V20Gy/V40Gy/V50Gy/V60Gy esophagus: volume ($cm^{3)}$ of the esophagus received at least 20/40/50/60 Gy; PTV: planning target volume; WHO: world health organizationRadiotherapy; technique: full volumetric modulated arc therapy.

a

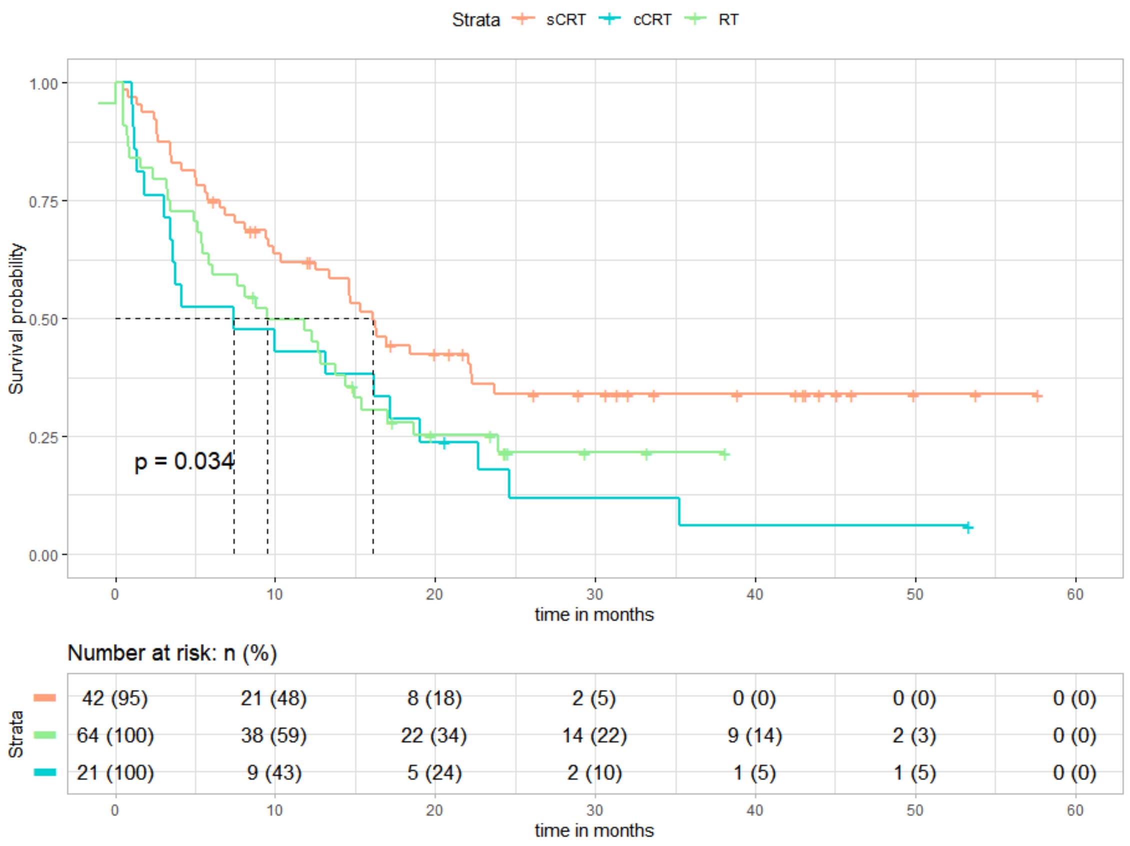

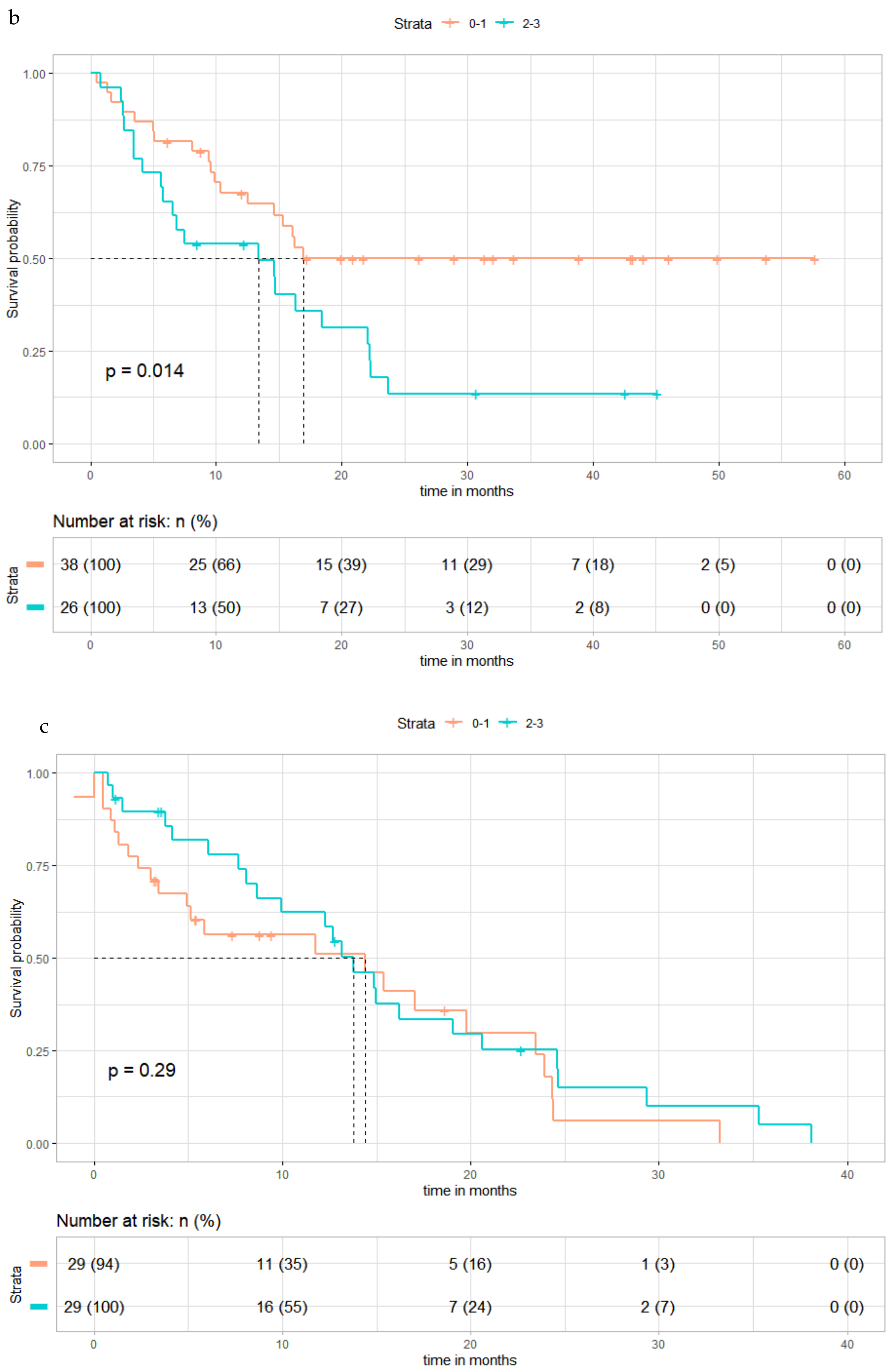

Figure S1. (**a**) Kaplan-Meier curves for progression free survival (PFS) for cCRT, sCRT and RT, (**b**) Kaplan-Meier PFS curves for patients with esophagitis grade 0–1 and grade 2–3, for cCRT only, (**c**) for sCRT/RT.
